# Preclosure spectroscopic differences between healed and dehisced traumatic wounds

**DOI:** 10.1371/journal.pone.0204453

**Published:** 2018-09-27

**Authors:** Jason S. Radowsky, Romon Neely, Jonathan A. Forsberg, Felipe A. Lisboa, Christopher J. Dente, Eric A. Elster, Nicole J. Crane

**Affiliations:** 1 Department of Surgery, Uniformed Services University of the Health Sciences and Walter Reed National Military Medical Center (USUHS-Walter Reed Surgery), Bethesda, Maryland, United States of America; 2 Orthopaedics, USUHS-Walter Reed Surgery, Walter Reed National Military Medical Center, Bethesda, Maryland, United States of America; 3 Orthopaedics, Johns Hopkins University, Baltimore, Maryland, United States of America; 4 Regenerative Medicine Department, Naval Medical Research Center, Silver Spring, Maryland, United States of America; 5 Surgical Critical Care Initiative, Uniformed Services University of the Health Sciences, Bethesda, Maryland, United States of America; 6 Department of Surgery, Emory University School of Medicine, Atlanta, Georgia, United States of America; 7 Trauma/Surgical Critical Care, Grady Memorial Hospital, Atlanta, Georgia, United States of America; Louisiana State University Health Sciences Center, UNITED STATES

## Abstract

**Background:**

The complexity and severity of traumatic wounds in military and civilian trauma demands improved wound assessment, before, during, and after treatment. Here, we explore the potential of 3 charge-coupled device (3CCD) imaging values to distinguish between traumatic wounds that heal following closure and those that fail. Previous studies demonstrate that normalized 3CCD imaging values exhibit a high correlation with oxygen saturation and allow for comparison of values between diverse clinical settings, including utilizing different equipment and lighting.

**Methods:**

We screened 119 patients at Walter Reed National Military Medical Center and at Grady Memorial Hospital with at least one traumatic extremity wound of ≥ 75 cm^2^. We collected images of each wound during each débridement surgery for a total of 66 patients. An in-house written computer application selected a region of interest in the images, separated the pixel color values, calculated relative values, and normalized them. We followed patients until the enrolled wounds were surgically closed, quantifying the number of wounds that dehisced (defined as wound failure or infection requiring return to the operating room after closure) or healed.

**Results:**

Wound failure occurred in 20% (19 of 96) of traumatic wounds. Normalized intensity values for patients with wounds that healed successfully were, on average, significantly different from values for patients with wounds that failed (p ≤ 0.05). Simple thresholding models and partial least squares discriminant analysis models performed poorly. However, a hierarchical cluster analysis model created with 17 variables including 3CCD data, wound surface area, and time from injury predicts wound failure with 76.9% sensitivity, 76.5% specificity, 76.6% accuracy, and a diagnostic odds ratio of 10.8 (95% confidence interval: 2.6–45.9).

**Conclusions:**

Imaging using 3CCD technology may provide a non-invasive and cost-effective method of aiding surgeons in deciding if wounds are ready for closure and could potentially decrease the number of required débridements and hospital days. The process may be automated to provide real-time feedback in the operating room and clinic. The low cost and small size of the cameras makes this technology attractive for austere and shipboard environments where space and weight are at a premium.

## Introduction

Despite many recent advances in wound care techniques, decisions to primarily close open wounds having undergone surgical débridement are still based on principles that have not changed for the past century. Called the four ‘C’s’, these include contractility of muscle when stimulated, color, consistency, and capacity to bleed.[1] However, while successful in a majority of cases, approximately 20% of wounds during the recent wars in Afghanistan and Iraq failed following primary closure.[2–4] This represents a significant expenditure in resources both in terms of consumable wound care supplies as well as increasing operating room utilization and hospital length of stay, not to mention prolonged patient discomfort and delay of rehabilitation therapies.

Because of this, considerable effort is being directed toward finding more objective parameters to enhance the surgeon’s decision-making capabilities. [2–9] These parameters include serum and wound cytokine analysis, such as the Department of Defense’s Surgical Critical Care Initiative wound biomarkers program, and data modeling algorithms which employ both clinical and laboratory values in order to predict wound failure. In this study, we expand the patient population to include civilian and military trauma patients. Much of the previous research has been conducted on military wounded [2–9], most of whom belong to a different demographic—predominately young, active, previously healthy males—and who sustain different mechanisms of injury (blast) than the typical civilian trauma patient (blunt trauma). In addition, military wounds are likely to be contaminated with pathogens generally unseen in civilian patients.[10,11] A recent, related study comparing wound biomarker profiles for military and civilian trauma patients established a similar pattern of systemic inflammation and wound dehiscence rates.[3] While extremely promising, these efforts have limitations in terms of the time requirement and cost of performing the requisite assays and calculations. In this study, we explore another technique, multispectral imaging with a 3CCD camera, which we believe could be complimentary to standard biomarkers for predicting wound failure.

Recent advances in image processing allow for the immediate assessment of tissue viability, something the authors believe beneficial when treating large, heterogeneous extremity wounds. In fact, previous studies utilized a 3CCD (three charge-coupled device) camera to evaluate changes in oxygenation in red blood cells[12], vessels[13], ischemic limbs[14–16], during pediatric appendectomies[17], and in partial and donor nephrectomies[18,19]. The present study seeks to ascertain the imaging characteristics of a heterogeneous population of extremity wounds prior to closure using a 3CCD camera, a commercially available and inexpensive device that records the levels of each of the three primary colors (red, green, and blue) to form a high-resolution image. We hypothesize that preclosure image values of the three primary colors will differ between wounds that fail and those that heal successfully following primary closure in correlation with the level of oxygen saturation of the wound surface and adjacent wound margins. Using this camera, we propose to quantify the visible light spectrum differences of these extremity wounds in both civilian and military trauma patients.

## Materials and methods

### Traumatic wound clinical studies

Following institutional review board (IRB) approval, we collected images from two locations–Emory Hospital/Grady Memorial Hospital in Atlanta, GA and Walter Reed National Military Medical Center (WRNMMC) in Bethesda, MD. As this was a study involving humans, the institutional IRBs set strict ethical standards. Between the two sites, we screened 119 patients for inclusion into this study. Of these, 39 declined participation and 14 were screen failures (6 in police custody, 6 unable to consent, 2 with language barriers), leaving 66 study participants who provided consent for the study, of whom 48 were from Emory Hospital/Grady Memorial Hospital and 18 from WRNMMC. All participants, or their designated representatives, provided written consent as approved by the two institutions’ IRBs. We photographed each wound at the index (initial) surgical débridement following admission or transfer to the study hospitals as well as during each subsequent surgical débridement leading up to wound closure; no photography was performed following wound closure. The number of surgical débridements for each wound varied; wounds were surgically closed at the sole discretion of the surgeon, as per standard of care. A total of 258 images were analyzed for this study, each débridement serving as an independent measurement. Final wound outcomes for each patient were recorded, healed or dehisced—respectively defined as either successful surgical wound closure (healed) or wound failure or infection after closure necessitating a return to the operating room (dehisced). Some healed wounds were also subclassified as delayed healing[20], meaning surgical wound closure occurred after more than 21 days post-injury. The demographics of the patient population at both institutions is listed in Table 1.

**Table 1:** Patient demographics by institution (Emory/Grady versus WRNMMC).

	Emory/Grady	WRNMMC	p-value
Number of Total Wounds	68	30	
Number of Wounds/Patient	1.6 ± 0.65	2.5 ± 1.2	*0*.002*
Mean Débridements/Wound	5.5 ± 3.3	5.6 ± 1.4	*0*.*19*
Mean Initial Wound Size (cm^2^)	255 ± 299	193 ± 133	*0*.*61*
Mean Age	38 ± 14	25 ± 9	*0*.000*
% Male	79.4	100.0	*0*.002*
% Female	20.6	0.0	*0*.002*
% Healed	85.3	67.9	*0*.035*
% Dehisced	14.7	32.1	*0*.035*

Values shown are mean ± standard deviation, except wound size (mean ± standard error of mean).

* = p-value ≤ 0.05.

### 3CCD image collection

The images were collected with a 3CCD camera (HDC-HS9, Panasonic, Chesapeake, VA, USA) following a standard operating procedure to ensure appropriate camera white balancing prior to image acquisition. Briefly, for 3CCD imaging, a color image is reconstructed and recorded using red (R), green (G), and blue (B) bandpass filters in front of three separate monochrome CCDs. The individual colors can be combined, subtracted and otherwise manipulated to enhance the contrast of an image so that detection is sensitive to molecules of interest. The contrast enhancement derived from 3CCD imaging stems directly from the absorption spectrum of oxygenated and deoxygenated hemoglobin and/or myoglobin.[12] Images were saved as JPEGs prior to image analysis.

### Data analysis

Prior to any analysis, all collected images that did not meet quality criteria (i.e. due to excessive glare or blood in the field of view, or image blurriness) were disregarded. Each image was analyzed utilizing an in-house written MATLAB (MathWorks, Natick, MA, USA) script that separated the images into those corresponding to the R, G, and B channels. Next, one or more regions of interest (ROI) within the image were selected and mean values and standard errors of mean were extracted for the ROIs. Mean ROI values for the R, G, and B channels were normalized to the mean R, G, and B channel value for each hospital (i.e. Emory/Grady or WRNMMC). This normalization step is critical for comparison of values between different operating rooms. Composite values such as R-B (“red minus blue”) were calculated by subtracting the mean ROI value extracted from the red channel from the mean ROI value extracted from the blue channel. A total of 14 imaging variables were extracted from each image: R, G, B, R-B, R-G, (R-B)/G, (R-B)/(R^2^+B^2^), and normalized values of the aforementioned variables. Note, for variable normalization, each variable was divided by the overall mean of each variable.

We used the Mann-Whitney U test to compare differences between outcomes (healed versus dehisced) for non-normally distributed continuous data. The levels of significance for the imaging data was adjusted using the false discovery rate method.[2] A chi-squared statistic was used to compare rates of wound dehiscence between the two cohorts. P-values less than 0.05 were considered statistically significant and are indicated by an asterisk (*). We used IBM SPSS v.24 (IBM Corp, Armonk, NY) and MATLAB for all statistical calculations.

Finally, a hierarchical cluster analysis (HCA) model to predict wound dehiscence was created based on two partial least squares discriminant analyses (PLSDA) models (PLS Toolbox, Eigenvector Research Inc., Manson, WA). The HCA model was trained and cross validated with a stratified, random subset that comprised 75% of the entire sample set. Stratification of the dataset ensures similar rates of wound outcomes for both the training and validation datasets, i.e. both subsets contained 20% wound dehiscence. The final validation of the model utilized a stratified external dataset consisting of the remaining 25% of the original data. The 17 variable PLSDA and HCA models were calculated using wound size, days post-injury, age, and fourteen 3CCD values (individual channels–R, G, B, and derivations/computations thereof). Lack of model overfitting was confirmed with permutation tests.[21,22] Briefly, the permutation tests measure the “probability of insignificance”; a p-value less than 0.05 prognosticates that the model is significantly different from a randomly generated model (95% confidence), and thus is not overfit.

## Results

### Comparing patients by institution and outcome

Approximately one third of the images were collected at WRNMMC and the remaining images were collected at Emory/Grady. Patient demographics compared by institution are presented in Table 1. The mean number of débridements per wound for civilian trauma patients and military trauma patients are similar (5.5 ± 3.3 vs. 5.60 ± 1.4, respectively; p = 0.19). It is notable, that the mean age of the civilian trauma patient is significantly higher than the mean age of the combat trauma patient (38 ± 14 vs. 25 ± 9, respectively; p < 0.001), and the percentage of female civilian trauma victims is higher than that of military trauma victims (20.6% vs. 0%, respectively; p = 0.002). The rate of dehiscence was lower in the civilian population (14.7% vs. 32.1%, respectively; p = 0.035) as was the number of wounds per patient (1.6 ± 0.65 vs. 2.5 ±1.2, respectively; p = 0.002). We observed no significant difference between the mean initial wound size between military and civilian patients (measured during the first débridement).

A comparison of wound and patient demographics by outcome rather than institution, displayed in Table 2, reveals that there is no statistically significant difference between the mean number of wounds per patient and the mean number of débridements per wound. Furthermore, the mean initial wound size (or surface area), measured during the first débridement following study enrollment, was not significantly different between wounds that healed and wounds that dehisced. Finally, the mean age of patients with healed wounds is not significantly different than the mean age of the patients with dehisced wounds.

**Table 2:** Comparison of wound and patient demographics by outcome (healed versus dehisced) for both institutions.

	Healed	Dehisced	p-value
Number of Wounds per Patient	1.81 ± 0.12	1.93 ± 0.30	*0*.*99*
Number of Débridements per Wound	5.46 ± 0.36	5.56 ± 0.65	*0*.*26*
Mean Initial Wound Size (cm^2^)	231 ± 30	298 ± 98	*0*.*92*
Mean Age	36.4 ± 2.1	36.3 ± 3.6	*0*.*69*

Values shown are mean ± standard deviation, except wound size (mean ± standard error of mean).

Lastly, a comparison of proportions of mechanism of injury for healed and dehisced wounds is listed in Table 3, described as percentages of the total healed and dehisced population. Mechanisms of injury include blast, gunshot wound (GSW), motor vehicle crash (MVC) pedestrian/vehicle strike, motorcycle accident, crush, falls >15 feet, falls <15 feet, and other. Chi-squared tests revealed statistically significant differences between healed and dehisced wounds for blast, GSW, MVC, and motorcycle accident injuries. For blast injuries, MVC, and motorcycle accidents, a larger proportion of wounds dehisced. For GSW and pedestrian-struck injuries, a smaller number of wounds dehisced. Interestingly, purely penetrating wounds accounted for 34% of the total wounds but failed only 6% of the time. Blunt injuries and combined (eg. blast injuries) failed 30% of the time, with the 9 of the 24 blast injuries dehiscing (37.5%). Flaps or skin grafts were used for 41% of the wound closures at Emory and 34% of the wounds at WRNMMC.

**Table 3:** Comparison of total wound outcome proportions by mechanism (healed versus dehisced).

Mechanism	Healed (%)	Dehisced (%)	p-value
Blast	19.2	47.4	*0*.01*
GSW	39.7	10.5	*0*.02*
MVC	3.8	15.8	*0*.05*
Pedestrian	12.8	5.3	*0*.*36*
Motorcycle	2.6	21.1	*0*.00*
Other	6.4	0.0	-
Crush	7.7	0.0	-
Fall >15 ft	3.8	0.0	-
Fall <15 ft	3.8	0.0	-

Chi-squared tests could not be calculated for Other, Crush, and Falls.

*A p-value ≤ 0.05 indicates statistical significance.

### Examining differences in 3CCD image values

**Fig 1:**
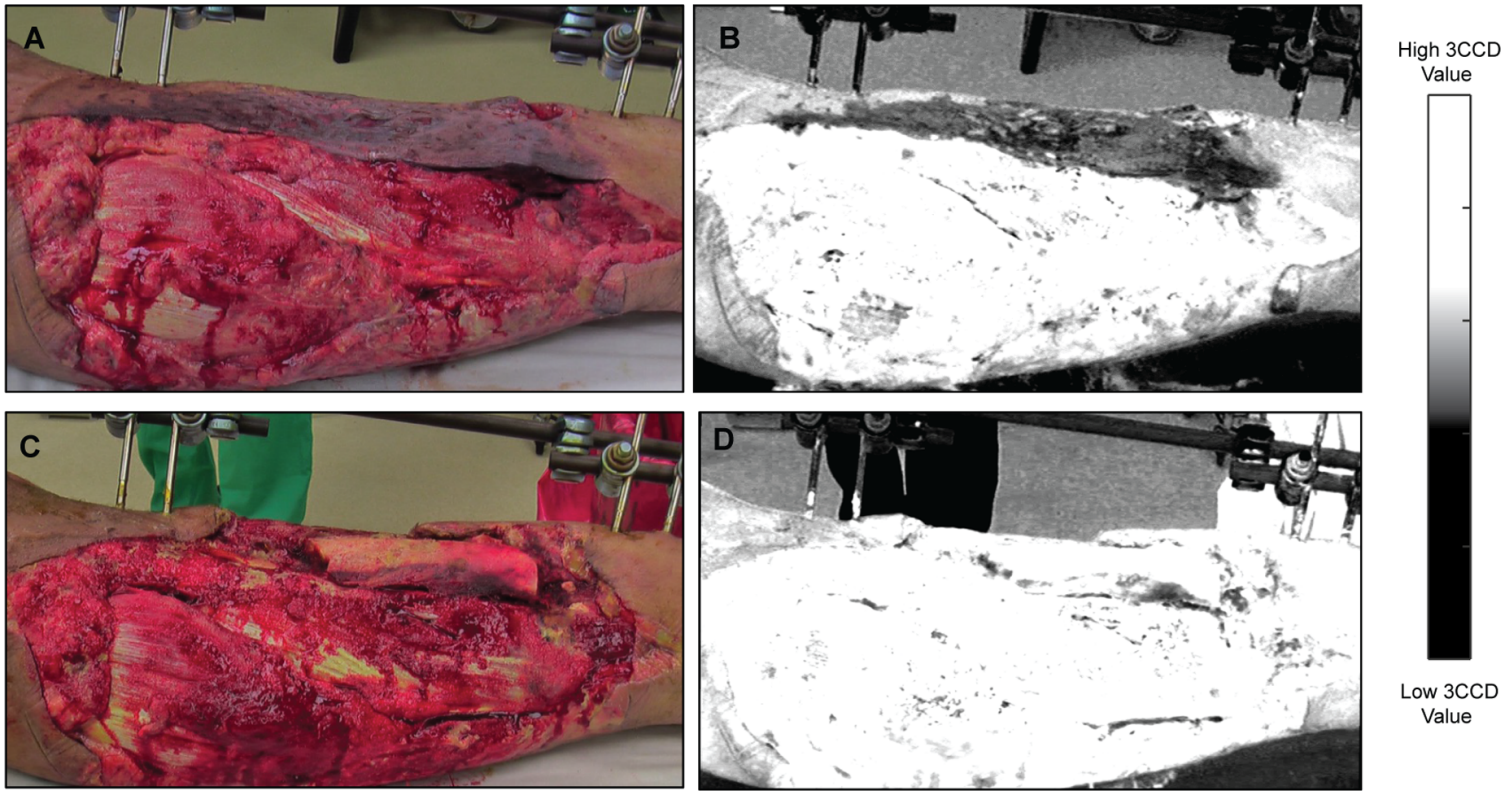
Regular 3CCD images (color) collected during a civilian trauma case (Emory/Grady) and the corresponding contrast enhanced images (grayscale); in this case, the wound healed normally. First débridement (A) from a healed wound and its corresponding contrast enhanced image (B). Last débridement (C) from the same case and the corresponding enhanced image (D). White regions have a higher 3CCD value while black regions have a lower 3CCD value given the fewer photons captured in that color spectrum by the camera. Note, there is little or no difference between the first and final débridement 3CCD values of the wound bed.

**Fig 2:**
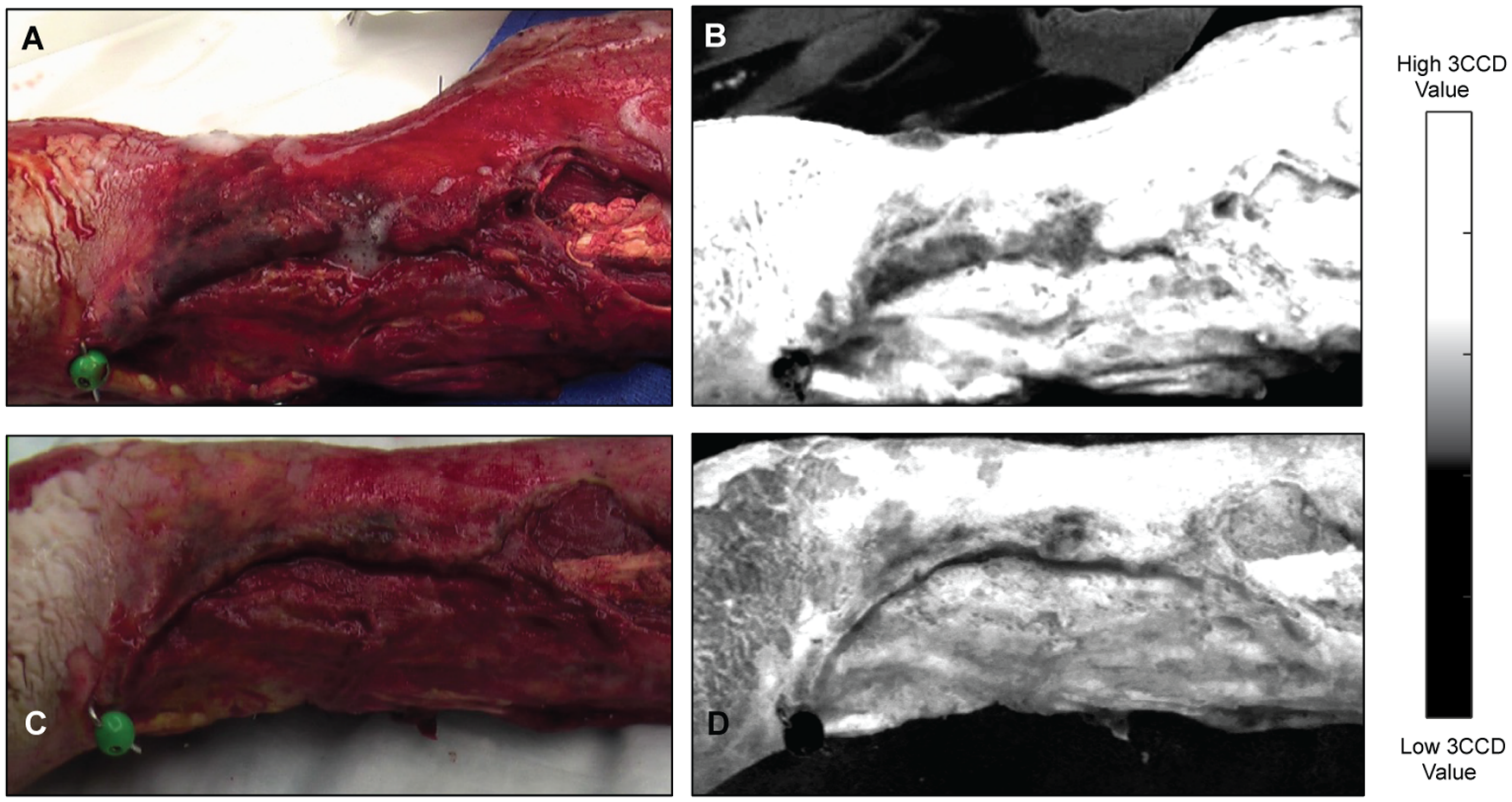
Regular 3CCD images (color) collected during a civilian trauma case (Emory/Grady) and the corresponding contrast enhanced images (grayscale); in this case, the wound dehisced. First débridement (A) from a healed wound and its corresponding contrast enhanced image (B). Last débridement (C) from the same case and the corresponding enhanced image (D). Notably, the wound bed 3CCD values are decreased at the last débridement (darker in color) when compared to the first débridement (lighter in color).

An example of a healed wound is shown in Fig 1. The images in Fig 1A and 1C correspond to the 3CCD images of a healed wound, where Fig 1A is an image of the first débridement and Fig 1C is an image of the final débridement. The matched, contrast enhanced images are displayed in Fig 1B and 1D. Similarly, the images in Fig 2 correspond to a dehisced wound.

**Fig 3:**
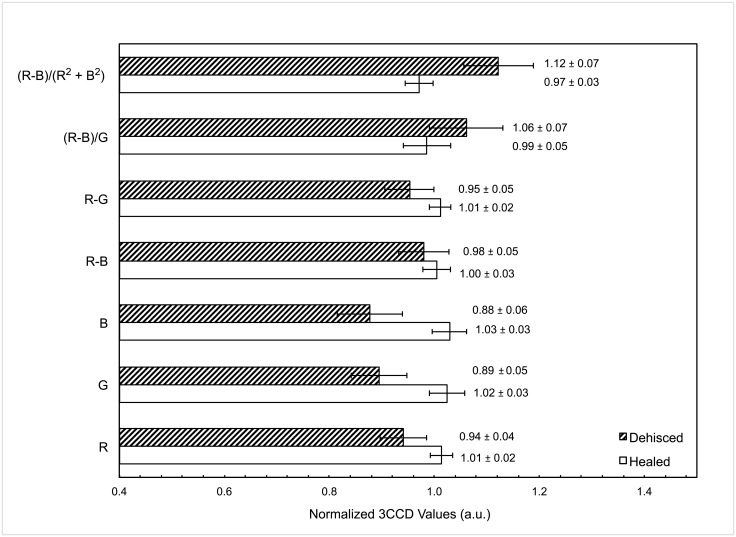
3CCD values for healed (white bars) and dehisced (striped bars) wounds with mean and standard error of the mean. Adjusted p-values for the imaging variables are 0.123, 0.178, 0.091, 0.854, 0.194, 0.112, and 0.112 for normalized R, G, B, R-B, R-G, (R-B)/G, and (R-B)/(R^2^+B^2^) values, respectively.

A comparison of the normalized 3CCD values between institutions revealed no statistically significant differences thus 3CCD values were pooled into a larger dataset of all trauma patients, both combat and civilian. Fig 3 depicts the differences in 3CCD values for healed and dehisced wounds. Individual R, G, and B channels show a statistically significant decrease in values for dehisced wounds compared to healed wounds. While the R-B and R-G values show no statistically significance difference between healed and dehisced wounds, these values are in general lower for dehisced wounds compared to healed wounds. The last two 3CCD values, (R-B)/G values and (R-B)/(R^2^ + B^2^) values, show the inverse trend and are significantly increased for dehisced wounds compared to healed wounds. Both the (R-B)/G values and (R-B)/(R^2^ + B^2^) values serve as means of normalization; for instance, dividing R-B by (R^2^ + B^2^) is a method by which to normalize intensity fluctuations of images and 3CCD values given user angle and distance, and institutional lighting variability during image capture.

### Predicting wound dehiscence with multivariate analysis

Attempts made to develop a basic (univariate) threshold model of the 3CCD values to predict wound outcome based on 3CCD values–where R-B/(R^2^ + B^2^) values ≥ 1.00 were assigned to dehisced wounds and R-B/(R^2^ + B^2^) values <1.00 were assigned to healed wounds, performed with poor accuracy (≤ 65%) and a low diagnostic odds ratio (≤ 2.5). Thus, multivariate models for prediction of wound outcome were explored.

**Fig 4:**
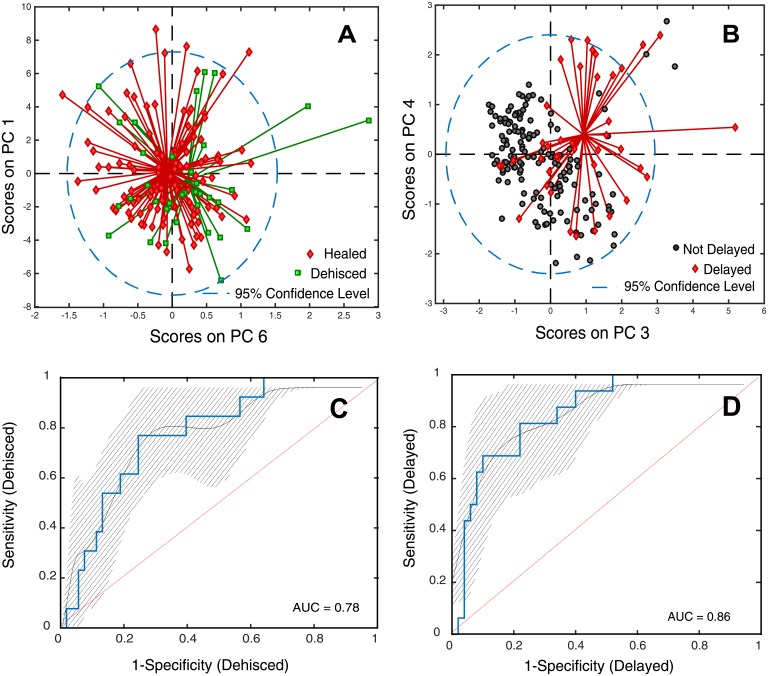
Comparison of principal component analysis (PCA) and partial least squares discriminant analysis (PLSDA). PCA score plots are shown for A) healed versus dehisced wounds, and B) delayed versus not-delayed wounds. There is significant overlap for healed and dehisced wounds while delayed and not-delayed wounds display some separation. Prediction ROCs derived from PLSDA models are exhibited with 95% confidence intervals in panels C&D (respectively) for: healed versus dehisced wounds, and delayed versus not-delayed wounds. The AUCs indicate improved separation of delayed and not-delayed wounds compared to healed and dehisced wounds.

Principal component analysis (PCA) can be used to reduce dataset noise and exploit class variance; here, PCA is used to visualize the data classes and any inherent separation of samples based on their wound healing outcome (i.e. healed versus dehisced). PCA score plots showed no discernable difference in clustering of healed and dehisced wounds (Fig 4A). However, when considering delayed healing wounds (surgical closure that was performed >21 days post-injury) versus not-delayed healing wounds (wounds that dehisced, or healed wounds that were surgically closed in <21 days post-injury), the delayed healing wounds demonstrate separation from all not-delayed healing wounds (Fig 4B).

In some cases where PCA does not perform well to distinguish classes, class separation can be achieved with partial least squares discriminant analysis (PLSDA). Here, two PLSDA models were generated and combined into a hierarchical cluster analysis (HCA) model, utilizing training and validation data sets. The first model maximized the separation between wounds subclassified as delayed healing or not-delayed healing (84.8% accuracy, diagnostic odds ratio of 19.8 with 95% confidence interval (CI) of 4.9–80.6). The second model took the remaining data and magnified the separation between wounds classified as healed and dehisced (72.7% accuracy, diagnostic odds ratio of 8.4 with 95% CI of 2.0–35.0). The corresponding prediction receiver operating curves (ROCs) are displayed, along with the AUCs in Fig 4C&4D, respectively. Lastly, in an effort to improve the model’s predictive performance, the HCA model performed the PLSDA models sequentially to classify wounds as healed or dehisced. While the final HCA model utilized three outcomes (healed, delayed, and dehisced), both healed wounds and delayed healing wounds were classified as a healed wound. The HCA model of the healed versus dehisced wounds demonstrated fairly accurate discrimination between the two cohorts (76.6% accuracy) with a diagnostic odds ratio of 10.8 (95% CI of 2.6–45.9). Type or etiology of the wounds did not assist in the model accuracy. Closure type was not included in the model either.

## Discussion

Non-invasive 3CCD imaging, combined with multivariate statistical analysis, provides the means to distinguish healed and dehisced wounds with greater than 75% sensitivity, specificity, and accuracy. This study may lead to novel strategies for clinical decision-making in the future. Not only would models based on these differences be beneficial for deciding whether or not to close wounds, but may also provide information as to whether or not a certain therapy was working from one débridement to the next by revealing the trajectory of 3CCD values. This possibility is clearly illustrated with contrast enhanced 3CCD images in Figs 1 and 2. To the naked eye, there is very little difference between the image from the first and the final débridements for the healed wound (Fig 1). However, the dehisced wound (Fig 2) demonstrates noticeably lower contrast enhanced values in the area of the wound for the final débridement compared to the healed wound.

Three-charged coupled device cameras hold significant advantages over other available technologies which measure tissue oxygenation or blood flow. First, it is a well-established imaging device that is available commercially from a number of electronics manufacturers and is very inexpensive compared to most medical imaging apparatuses; running about $1,000 USD depending on device. Second, it is a technology that can be incorporated into small wireless devices, such as cellular phones and tablets, making it extremely portable. In 2015, Apple was granted a U.S. patent for a three-sensor based camera within the small form factor of a wireless device, such as an iPhone.[23]

Other technologies, such as indocyanine green fluorescence imaging, laser doppler flowmetry, laser speckle contrast imaging, harmonic ultrasound imaging, and hyperspectral imaging assess wound healing characteristics such as wound perfusion, wound depth, and oxygen saturation.[24–31] However, all require expensive equipment and ancillary purchases like injectable contrast agents that have anaphylactic potential and have storage requirement conditions. Few studies exist where true hyperspectral imaging has been employed continuously and in real-time. In addition, many of these technologies cannot generate models that can be adapted to portable wireless devices, severely limiting their distribution. The combination of the low cost of three-sensor camera technology, transportability of small form factor wireless devices, and simplicity of model implementation makes 3CCD imaging an ideal candidate for austere and shipboard medical environments where space, weight, and sustainment logistics are at a premium.

The similarity between the normalized 3CCD values between the WRNMMC and Emory/Grady wounds indicates that this modality is beneficial for both civilian and military traumatic wounds. This is important given the different mechanisms (blasts, or other high energy mechanisms in the military), along with the differences in patient demographics between the two cohorts. As such, these results suggest that 3CCD imaging may be generalizable to both patient populations. This is consistent with our previously reported findings, that despite different injury mechanisms, biologic responses are similar in military and civilian extremity injury patients.[7] Furthermore, in larger cohorts of related studies[3], similar rates of dehiscence are observed, which speaks to the generalizability of our findings. In the larger military patient cohort, the mean wound dehiscence rate was 13% less than presented in this study; our inflated wound dehiscence rate for military patients in this study can be attributed to sample size–the larger study includes 116 wounds while our study includes 30 wounds.

In previous studies, increases in R-B values indicated increased oxygenation of the wounds through more robust microvasculature.[14–16] This is likely secondary to the increases in angiogenesis in wounds that heal compared with those that dehisce. In this study, however, we had to explore more robust normalization techniques to exploit the small differences in the R-B values between healed and dehisced wounds. Dividing the R-B values by (R^2^ + B^2^) simply magnifies the percent difference between R-B values for healed and dehisced wounds. In fact, the differences between the (R-B)/(R^2^ + B^2^) values for healed and dehisced wounds is the largest and most significant of all imaging parameters investigated. With both cohorts, decreases in the R-B/(R^2^ + B^2^) values were demonstrated by healing wounds. However, using a threshold R-B/(R^2^ + B^2^) value performed unsatisfactorily to discriminate dehisced wounds from healed wounds, and with poor accuracy (<65%).

Increased (R-B)/(R^2^ + B^2^) values in dehisced wounds alone are not enough to portend wound failure and initial multivariate PLSDA models that integrated all 3CCD variables performed with less than 75% accuracy, sensitivity, and specificity. Data exploration with principal component analysis flaunted the fact that there was little separation between healed and dehisced wounds, and exposed the need to include a healed wound subclassification: delayed healing. This additional category (delayed versus not-delayed), when accounted for in the PLSDA-based HCA model, improved specificity and accuracy to > 75%. While PLSDA models are prone to overfitting data, here we confirmed that the models were appropriately fit by utilizing permutation tests (similar to random forest shuffling).[32] Two advantages of using PLSDA models is their simplicity and speed, which make them easy to implement real-time and easy to update as new data is collected.

The results depicted in this study need to be considered with the weight of its limitations. First, the statistical analysis and modeling of spectrographic data was performed *post-hoc*, in a controlled setting, and is a limitation of this study. Nevertheless, this theoretical limitation could be mitigated by implementing on-chip code, designed to provide real-time analysis that is embedded in the camera hardware itself and system standardization (to account for user and institutional variability). Next, the data is not distributed evenly between military and civilian patient populations– 31% combat-related trauma wounds versus 69% civilian trauma wounds. While there are some inherent differences between military and civilian trauma, preliminary analysis and comparison of the imaging and clinical data extracted for both military and civilian patients showed no statistically significant difference aside from age and gender, so patients were pooled into a larger trauma cohort. It is expected that the military population in this study and related studies is generally a more homogenous study population (young, healthy men). In spite of this, a related study demonstrated similarities in systemic inflammatory profiles of military and civilian trauma patients.[3]

We believe the encouraging results warrant further investigation with larger enrollment–particularly combat-induced wounds; this may generate an even more robust model to be validated in a prospective manner. Lastly, a larger study cohort would also enable the study of imaging variables chronologically, which may provide insight to wound healing mechanisms such as angiogenesis. Furthermore, including culture data to ascertain bacterial or fungal colonization or frank infection of wounds would be a welcome addition to future study data as this information was not included in this cohort. The ability to predict wound dehiscence during the course of treatment may provide an opportunity to reduce not just the cost of caring for the critically ill, but also reduce days spent in the ICU and rehabilitation (1.7 days and 4.2 days, respectively). A 5% reduction in wound dehiscence rate in traumatic extremity wounds has been estimated to be equivalent to a cost savings of approximately $1 billion dollars (USD).[3]

Thus, this would assist the surgeon in deciding whether or not to close the wound at the time of operation by augmenting the subjective clinical evaluation of the wound, and potentially saving patients unnecessary further débridement, wound failure, and prolonged hospitalization with the resulting metabolic and physiologic insults. Thus, this technology might allow for precision surgical care by alerting to the surgeon to the appropriateness of the wounds for closure. This is even more important for flap and skin-graft closures as another wound is being created in the attempt to close the injury and thus even more of the patient’s metabolism and physiologic reserve is invested in the closure. As a high proportion of the wounds in this study were closed in this manner, it is encouraging to see the application of 3CCD cameras to this field.

In conclusion, the results of this study justify validation studies in larger civilian and military trauma patient populations. Additional data could be used to elucidate relationships beyond what the naked eye is able to detect, for example identifying wound healing stages or identifying wound complications such as infection or compartment syndrome. Moreover, this technology may serve as a component in a decision support algorithm, which would incorporate serum and wound effluent analysis as well as clinical information.[3] The potential telemedical applications of this technology for use in austere and shipboard environments should also undergo further evaluation.
